# TRIM68, PIKFYVE, and DYNLL2: The Possible Novel Autophagy- and Immunity-Associated Gene Biomarkers for Osteosarcoma Prognosis

**DOI:** 10.3389/fonc.2021.643104

**Published:** 2021-04-22

**Authors:** Jie Jiang, Dachang Liu, Guoyong Xu, Tuo Liang, Chaojie Yu, Shian Liao, Liyi Chen, Shengsheng Huang, Xuhua Sun, Ming Yi, Zide Zhang, Zhaojun Lu, Zequn Wang, Jiarui Chen, Tianyou Chen, Hao Li, Yuanlin Yao, Wuhua Chen, Hao Guo, Chong Liu, Xinli Zhan

**Affiliations:** Department of Spinal Orthopedics, The First Clinical Affiliated Hospital of Guangxi Medical University, Nanning, China

**Keywords:** osteosarcoma, autophagy, immune infiltration, prognostic biomarkers, quantitative reverse transcription PCR, Immunohistochemistry

## Abstract

**Introduction:**

Osteosarcoma is among the most common orthopedic neoplasms, and currently, there are no adequate biomarkers to predict its prognosis. Therefore, the present study was aimed to identify the prognostic biomarkers for autophagy-and immune-related osteosarcoma using bioinformatics tools for guiding the clinical diagnosis and treatment of this disease.

**Materials and Methods:**

The gene expression and clinical information data were downloaded from the Public database. The genes associated with autophagy were extracted, followed by the development of a logistic regression model for predicting the prognosis of osteosarcoma using univariate and multivariate COX regression analysis and LASSO regression analysis. The accuracy of the constructed model was verified through the ROC curves, calibration plots, and Nomogram plots. Next, immune cell typing was performed using CIBERSORT to analyze the expression of the immune cells in each sample. For the results obtained from the analysis, we used qRT-PCR validation in two strains of human osteosarcoma cells.

**Results:**

The screening process identified a total of three genes that fulfilled all the screening criteria. The survival curves of the constructed prognostic model revealed that patients with the high risk presented significantly lower survival than the patients with low risk. Finally, the immune cell component analysis revealed that all three genes were significantly associated with the immune cells. The expressions of TRIM68, PIKFYVE, and DYNLL2 were higher in the osteosarcoma cells compared to the control cells. Finally, we used human pathological tissue sections to validate the expression of the genes modeled in osteosarcoma and paracancerous tissue.

**Conclusion:**

The TRIM68, PIKFYVE, and DYNLL2 genes can be used as biomarkers for predicting the prognosis of osteosarcoma.

## Introduction

Osteosarcoma is one of the most common malignancies among orthopedic tumors. In 2019, a study conducted on osteosarcoma survival and prognosis reported that all the patients with a median follow-up time of 54 months who were biopsied presented the 3-and 5-year event-free survival rates of only 59% and 54%, respectively (1). Osteosarcoma is diagnosed in less than 1% of all cancers each year and is reported to be significantly associated with cancer mortality and morbidity (2). Surgical resection continues to be an indispensable treatment option for osteosarcoma, and for patients with severe symptoms, systemic chemotherapy is used for controlling the metastases (3).

Autophagy is a mechanism of cell death, in which a cell engulfs its cytoplasmic proteins or organelles and encapsulates them into vesicles, where the degradation of the contents finally occurs. Interestingly, recent research has demonstrated the reversal of autophagy in cancer, depending on the context, and the inhibition of autophagy has been proposed as a novel approach to treat cancer (4). Moreover, autophagy is reported to have an integral role in the metastatic process of the tumor (5). Autophagy, as a dynamic biological system of recycling and degradation, can contribute to tumor survival and growth on the one hand, and to the aggressiveness of cancer cells by promoting their metastasis on the other (6). We selected the set of examined genes associated with autophagy from The Gene Set Enrichment Analysis (GSEA, https://www.gsea-msigdb.org/gsea/index.jsp) for follow-up analysis in order to investigate in depth the role of autophagy-related genes in osteosarcoma.

Infiltration of tumor immune cells is an important step in the development of tumors, which appear to respond by resisting this immune attack by suppressing the immune system (7). This immunosuppression in the tumor microenvironment results in the suppression of the function of CD8+ T cytotoxic T lymphocytes (CTLs), further promoting tumor development (8). Therefore, CTLs are the preferred immune cell type in targeted cancer therapies. It has been previously shown that anti-tumor immune responses can be enhanced by inducing innate and adaptive immune mechanisms that bind to tumor-targeting antibodies (9).

Tripartite Motif Containing 68 (TRIM68), a protein-coding gene, is associated with TRIM68 in diseases, including systemic lupus erythematosus and prostate cancer. With regard to TRIM68, there have been many studies showing that this gene is particularly strongly associated with cancer, especially in prostate cancer (10, 11). However, there are few reports on the relationship between TRIM68 and tumor immunity. Phosphoinositide Kinase, FYVE-Type Zinc Finger Containing (PIKFYVE), is a protein-encoded gene that is most closely related to the diseases such as fleck and corneal dystrophy. Interestingly, it has been shown that PIKFYVE plays an integral role in endometrial cancer in terms of autophagy (12). There are few reports on this gene and tumor immunity. Dynein Light Chain LC8-Type 2 (DYNLL2), a protein-encoded gene that is primarily associated with short-rib thoracic dysplasia 11 with or without polydactyly and Bardet-Biedl syndrome 7. For the moment, most of our research on DYNLL2 is in the non-oncology area (13, 14). The role of these genes in relation to tumor immunity and to autophagy in osteosarcoma is not known until now.

There is insufficient evidence for the genes associated with autophagy and tumor immune infiltration in osteosarcoma. Therefore, the present study was aimed to identify novel prognostic biomarkers for osteosarcoma that are associated with autophagy and immune cell infiltration. This was accomplished by analyzing the autophagy-related genes and tumor immune cell infiltration in osteosarcoma, investigating the relationship between these genes and osteosarcoma prognosis, and determining the role of immune cell infiltration in osteosarcoma. In turn, this will provide clinical guidance to predict the prognosis of patients with osteosarcoma and provide a target for immunotherapy of osteosarcoma.

## Materials and Methods

### Data Download

The gene expression and clinical information data were downloaded from the UCSC Xena database (http://xena.ucsc.edu/) for the osteosarcoma samples and from the GTEx database (https://www.gtexportal.org/home/) for the healthy samples. All the gene expression data were subjected to further analysis. The log2(x+1) conversion was performed prior to the analysis. The autophagy-related gene sets were downloaded from the GSEA database. All plots and statistical calculations for this study were performed using the programming language R software version 4.0.2.

### Differentially Expressed Gene Analysis and Functional Enrichment Analysis of Autophagy-Related Genes

The Limma package (15) was employed for the differentially expressed genes (DEGs) analysis of all the genes in the gene expression matrix, with logFC > 1 and FDR < 0.05 as the threshold values. The heat maps and volcano maps for the genes were generated using the edgeR, pheatmap, and ggplot2 packages. Subsequently, based on the autophagy-related genes provided in the GSEA database, the autophagy-related genes were extracted from the expression matrix and subjected to the differential expression analysis with logFC > 1 and FDR < 0.05 as the threshold values. Next, the clusterProfiler package (16), the org.H.eg.db package, the enrichplot package (16), and the ggplot2 and GOplot packages (17) were employed to perform the Gene Ontology enrichment analysis (GO) on the DEGs (18) and the KEGG enrichment analysis (KEGG) (19), considering P-value < 0.05 as the significance threshold. The first ten entries of GO and the first ten entries of KEGG pathway were visualized.

### Construction of a Prognostic Model for Osteosarcoma

First, the univariate Cox regression analysis was performed to determine the survival time and survival status of the patients. Next, the screened genes were analyzed using the following screening condition: the univariate Cox regression analysis yielding a P-value < 0.01. In the second step, the least absolute shrinkage and selection operator (LASSO) method was used to further improve the accuracy of the model by identifying the most critical genes for which the prediction accuracy could be significantly improved. Finally, the multivariate Cox regression analysis was used to screen the genes obtained in the previous step, with P-value < 0.05 as the screening condition. The genetic and risk scores for the prognostic model were obtained.

### Diagnostic Curve ROC Analysis

The ROC curves for the constructed model were generated and analyzed using the survival package, survwiner package, and timeROC package. The ROC curves for one year, two years, and 3 years were generated.

### Differential Analysis and Principal Components Analysis of the Model Genes of High- and Low-Risk Groups

The freshape2 package and the ggpubr package (https://github.com/kassambara/ggpubr) were used for the differential expression analysis of the genes that were used for constructing the model, based on high- and low-risk groups. The results were visualized in violin plots. In addition, the scatterplot3d package was used to perform the principal component analysis of the high-and low-risk groups predicted by the constructed model.

### Prognostic Analysis

The endpoint time used in the prognostic analysis in our study was patient death. Survival analysis was performed, and Kaplan-Meier survival curves were plotted for both groups using the survival package and the survminer package, classifying the patients into high-and low-risk groups based on the high-risk and low-risk prediction, respectively, by the model. Subsequently, the patients were divided into high-expression and low expression groups based on the high expression and low expression of a particular gene, followed by survival analysis of both the groups using the survival package and plotting of Kaplan-Meier survival curves based on the high and low expressions of a particular gene.

### Risk Curve, Survival State, and Risk Heat Maps

The pheatmap package was employed to analyze the risk profiles of all patients, and based on the risk scores, the model genes were ranked from lowest to highest. The risk-related risk profiles, risk survival status maps, and risk heat maps were plotted.

### Predicting the Probability of Survival of the Patients With Osteosarcoma

The rms package was used for predicting and testing the risk profile of the constructed model. A calibration chart was prepared to evaluate the accuracy of our model, while a line chart was used for predicting the patient’s risk.

### Estimation of the Proportion of Immune Cell Types and Immune Composition of Model Genes

In order to quantify the proportion of immune cells, the CIBERSORT algorithm was applied to estimate the proportion of immune cells in the expression matrix. CIBERSORT is a sophisticated tool for characterizing the percentage of cellular composition in the gene expression profiles (20). CIBERSORT utilizes Monte Carlo sampling to obtain the P-value of the deconvolution for each sample (21). Therefore, this method is one of the most reliable methods for estimating the content of immune cells. The samples for which a P-value of <0.05 was obtained were selected for further analysis. For each of these samples, the sum of the fractions of the immune cell types was 1. Subsequently, immune composition analysis was performed for the three genes used for constructing the model and based on these three genes, the immune cell composition of each sample was analyzed.

### Real-Time Quantitative Reverse Transcription PCR (qRT-PCR)

The normal human osteoblasts hFOB1.19, human osteosarcoma cells SJSA-1 and human osteosarcoma cells HOS used in this study were purchased from Shenzhen Aowei Biotechnology Co (http://www.otwobiotech.com/). We used normal human osteoblasts hFOB1.19 as normal control cells for osteosarcoma with reference to previous studies (22, 23). The cells were cultured in Dulbecco’s modified Eagle’s medium (DMEM) containing 10% FBS, 100 U/mL penicillin, and 100 mg streptomycin at 37°C and 5% CO_2_ atmosphere. RNA extraction using Hipure Total RNA Mini kit (Magen, China) followed by quantitative real-time PCR (qRT-PCR) was used to purify the total intracellular RNA from the induced samples. Subsequently, 1000 ng of the extracted RNA was reverse transcribed into cDNA using a cDNA synthesis kit (Takara, China). qRT-PCR was used to detect the gene expression using the LightCycler 480 Sequence Detention System (Roche, Germany) and PCR Green Master Mix (Roche, Germany). The activation cycle of the polymerase included 10 min at 95°C and 15 s at 95°C, and 45 cycles such cycles were performed. Glyceraldehyde 3-phosphate dehydrogenase (GADPH, Abcam, USA) was used as the internal control, and the data analysis was performed using the 2^–ΔΔCT^ method. The analysis for each gene was performed in triplicate. The primer sequences of the target genes are presented in **Table 1**.

**Table 1:** Forward and directional sequences of primers for TRIM68, PIKFYVE, DYNLL2, and GAPDH.

Primer	Sequence (5′ to 3′)
TRIM68-F	AGGGCCCTGACAACTCTTTT
TRIM68-R	GGGAGCCACAGTCAGTCACATTG
PIKFYVE-F	TGGACGTTGGCTGGATTGTGTTAG
PIKFYVE-R	TCACTGAGTCACTGTCGGGAGAAG
DYNLL2-F	AGACCCTGCCACATCTCCTATGC
DYNLL2-R	CCACTGCCACCATGCCAACC
GAPDH-F	CCACTCCTCCACCTTTGAC
GAPDH-R	ACCCTGTTGCTGTAGCCA

### Immunohistochemistry

We used tumor sections and paraneoplastic tissue sections, for immunohistological studies, from patients with osteosarcoma who underwent surgery at the First Clinical Affiliated Hospital of Guangxi Medical University. The study was approved by the Ethics Department of the First Clinical Affiliated Hospital of Guangxi Medical University, in accordance with the Declaration of Helsinki of the World Medical Association. We performed immunohistological analysis on six pairs (osteosarcoma and paraneoplastic tissues) of a total of 36 pathological sections for each of the three genes. Immunohistochemical staining was performed to verify the accuracy of our analysis for the genes used to construct the model based on osteosarcoma and paraneoplastic tissues. Antibodies for immunohistochemical analysis were purchased from Bioss (http://www.bioss.com.cn/index.asp, TRIM68, item number: bs-17123R; DYNLL2, item number: bs-14469R) and PIKFYVE was purchased from Proteintech (https://www.ptgcn.com/, item no. 13361–1-AP). We first dewaxed and hydrated the pathological tissue sections, then performed microwave repair of the antigen, used PBS buffer for closure, incubated the primary antibody for one hour at room temperature with moisturizing oscillation, followed by secondary antibody incubation, followed by amplification of the fluoroscopic staining signal, and finally sealed the pathological tissue sections with completed staining. Finally, the stained osteosarcoma and paraneoplastic tissue sections were placed under a microscope to observe their protein expression. We performed statistical analysis of the images from immunohistology studies using ImageJ software to calculate the positive rate of immunohistology images more precisely. Then, we performed statistical analysis of the immunohistology positive rate for osteosarcoma and the immunohistology image positive rate for paracancerous tissue samples using the paired sample mean t-test in IBM SPSS Statistics 25 software. The visualization of the positive rate of immunohistology was done by GraphPad Prism8.

## Results

### Data Download and Differentially Expressed Gene Analysis

**Figure 1:**
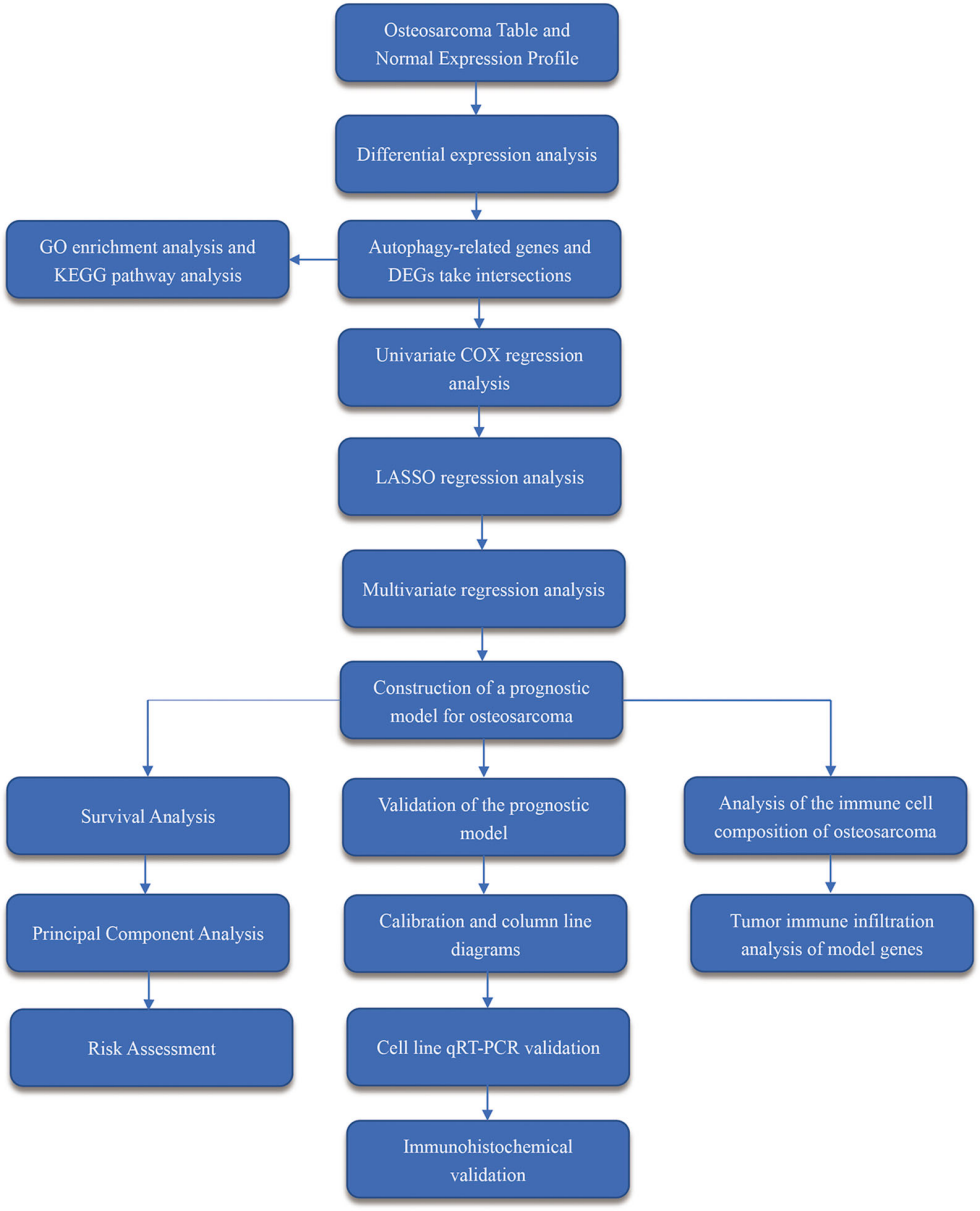
Workflow diagram. It shows the brief steps of all the procedures done in this study.

**Figure 2:**
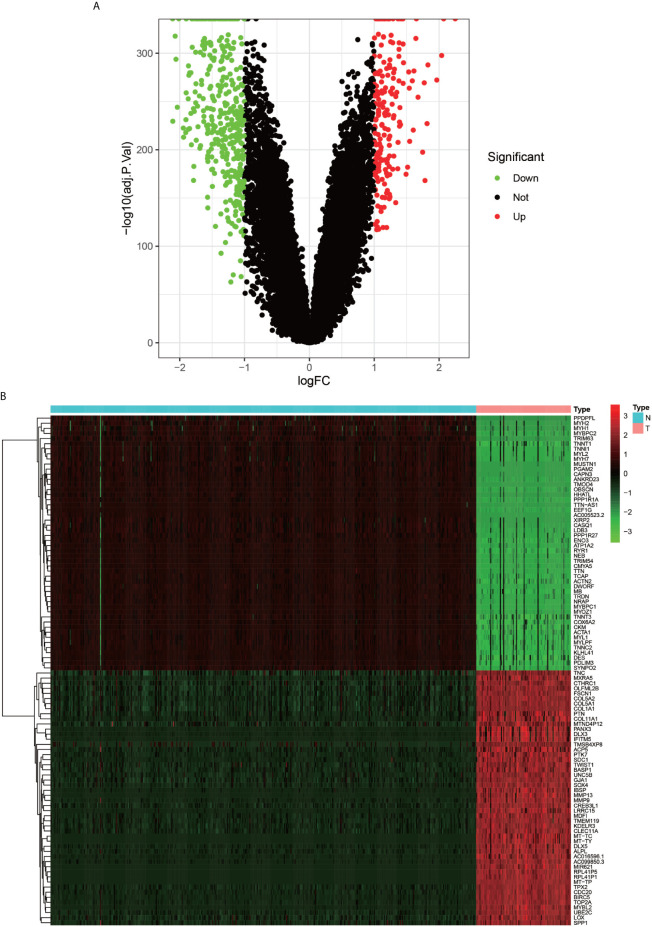
Volcano plot and Heat map of differentially expressed genes. **(A)** graph shows a volcano plot of DEGs, with red dots for high expression, green dots for low expression, and black dots for genes that do not meet our requirements. **(B)** graph shows a heat map of DEGs with normal samples in blue and tumor samples in red in the Type column; high expression genes in red and low expression genes in green in the graph.

The flow chart of the overall procedure is presented in **Figure 1**. A total of 88 osteosarcoma gene expression matrices along with their clinical data were downloaded from the UCSC Xena database, and the corresponding data for 396 healthy samples (normal controls) were downloaded from the GTEx database. In addition, 19 human autophagy-associated gene sets totaling 207 autophagy-associated genes from the GSEA database were downloaded. The differential analysis of the gene expression matrix comprising 88 tumors and 396 normal samples was performed for a total of 54,751 genes, which revealed 575 DEGs that were subsequently visualized as heat maps and volcano maps (**Figures 2A, B** **)**. The autophagy-related basal table matrix was extracted, and a difference-in-differences analysis was performed, which identified 194 upregulated autophagy-related DEGs.

### GO Enrichment Analysis and KEGG Pathway Enrichment Analysis

**Figure 3:**
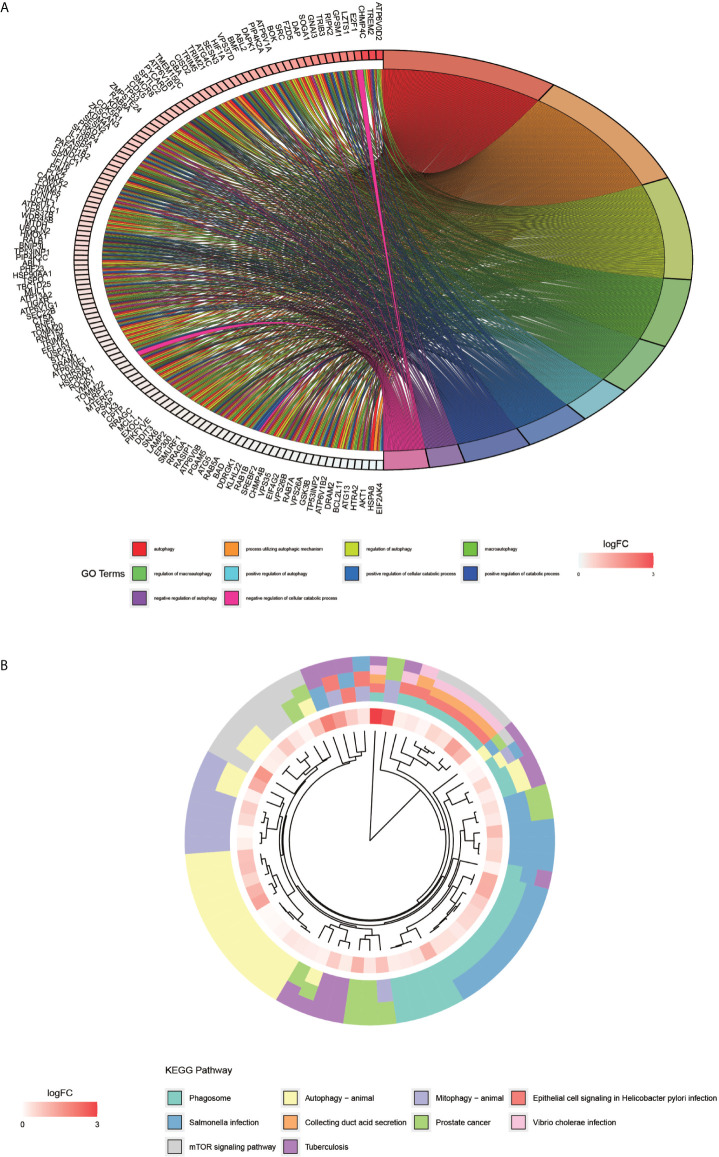
GO enrichment analysis of autophagy-related genes and KEGG pathway enrichment analysis. **(A)** graph shows the GO enrichment analysis of autophagy-related genes. The different color modules on the right side of the graph represent different GO entries; the left side represents genes, and the shades of red indicate the magnitude of the logFC values. **(B)**, showing the first ten entries of KEGG. The different color modules represent different KEGG pathways. The innermost color indicates the size of the logFC value of the gene.

The processing and analysis in the R software provided the results of the pre-GO enrichment analysis, among which, the top 10 entries (**Figure 3A**) were distributed mainly in autophagy, a process utilizing autophagic mechanism, and the regulation of autophagy. KEGG pathway (**Figure 3B**) was enriched mainly in the phagosome, autophagy-animal, and mTOR signaling pathways.

### Construction of a Prognostic Model for Osteosarcoma

**Figure 4:**
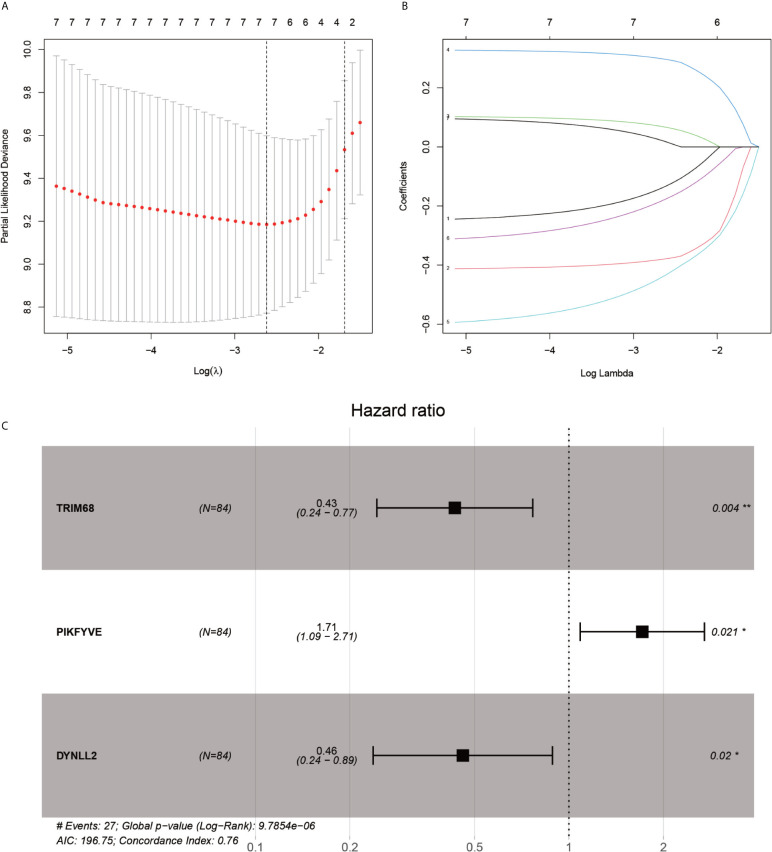
LASSO regression and multivariate COX regression analysis graph. **(A)**, Tenfold cross-validation of LASSO regression model adjustment parameter selection. **(B)**, LASSO coefficient curves for the seven analyzed genes included in the analysis. **(C)**, forest plot of multifactorial Cox regression analysis. P-value < 0.05 from multifactorial Cox regression analysis of TRIM68, PIKFYVE and DYNLL2. “*” represents P < 0.05, “**” represents P < 0.01.

After the first round of processing using the univariate Cox regression analysis (see **Table 2** for details), 7 out of the 207 autophagy-related genes fulfilling the screening criteria were obtained, which were then subjected to the LASSO regression analysis to improve further the accuracy of the model (**Figures 4A, B** **)**. Finally, a multivariate Cox regression analysis was performed, after which only three genes remained that fulfilled all our screening criteria: TRIM68, PIKFYVE, and DYNLL2 (**Figure 4C** **)**. Moreover, each sample was assigned a risk score and a high-or low-risk group.

**Table 2:** Results of univariate Cox regression analysis.

id	HR	HR.95L	HR.95H	p value
TIGAR	0.525575	0.309986	0.8911	0.016941
HGF	0.443967	0.223984	0.880001	0.02001
HMOX1	0.774269	0.610891	0.981343	0.034369
IL10RA	0.590749	0.372584	0.936662	0.025208
LZTS1	1.483641	1.020483	2.157009	0.038813
VMP1	0.502859	0.284441	0.888997	0.018045
MTDH	1.641143	1.003294	2.684507	0.048489
RALB	0.575841	0.347825	0.953333	0.031892
TP53INP2	0.526186	0.289284	0.957092	0.03541
TRIM21	0.530255	0.331526	0.84811	0.008109
TRIM38	0.536945	0.294042	0.980506	0.042966
TRIM68	0.390442	0.222287	0.685802	0.001067
TRIM8	1.962971	1.303364	2.956392	0.001246
ATP6V0E1	0.652017	0.435261	0.976715	0.038058
BOK	1.327933	1.013702	1.739572	0.039519
DRAM1	0.570489	0.333778	0.975074	0.040144
PIKFYVE	2.218923	1.392656	3.535417	0.000798
RETREG3	0.462095	0.219896	0.971059	0.041603
UFC1	2.116362	1.090131	4.108668	0.026765
CHMP4C	1.28951	1.051983	1.580667	0.014371
DYNLL2	0.323175	0.170046	0.614201	0.000565
TOMM20	1.599332	1.016693	2.515865	0.042195
TUBA1A	0.469068	0.295518	0.74454	0.001321
TUBB6	0.472131	0.264524	0.842677	0.011115
BMP2	1.343259	1.053546	1.712639	0.017275
CDC25B	0.550175	0.324441	0.932968	0.026592
IGF1R	1.349928	1.080065	1.687219	0.008369
IRF5	0.417498	0.201289	0.865942	0.018942
MAPK14	0.486007	0.239433	0.98651	0.045764

### Differential Analysis of High- and Low-Risk Groups of Model Genes and Principal Components Analysis

**Figure 5:**
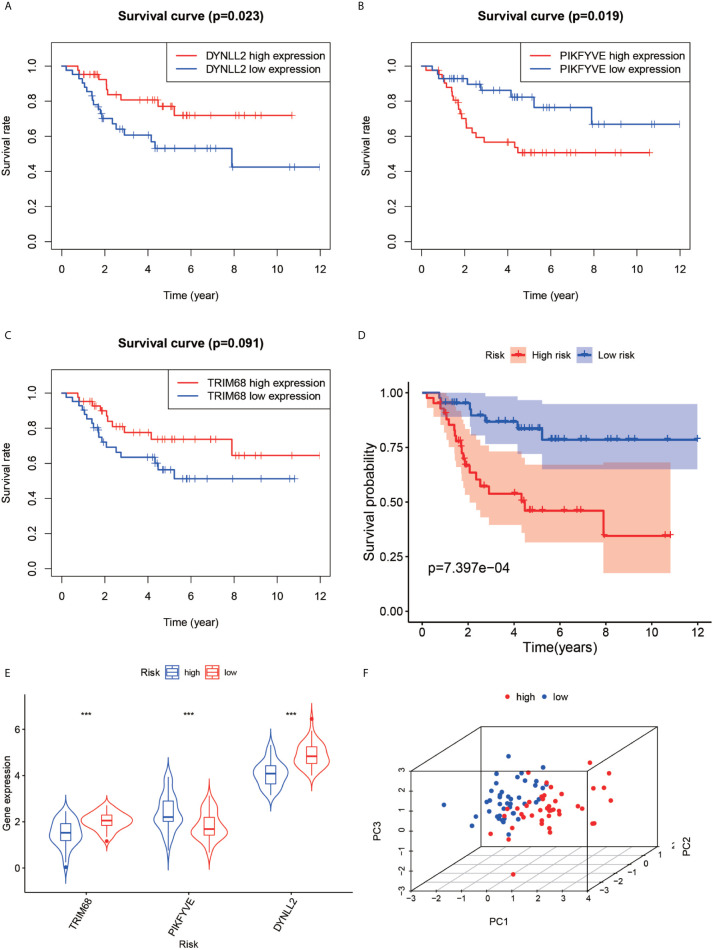
Survival analysis, violin plot and principal component analysis plot. Plots **(A–C)** show the survival analysis plots based on these three high and low gene expressions. Plots **(D)** shows the survival analysis plotted based on the high and low risk of the model. **(E, F)** show 3D display plots of gene expression and principal component analysis of the model for high and low risk, respectively. “***” Representative P < 0.001.

The violin plots were generated for the three selected genes (**Figure 5E**), which revealed that the median gene expression values in the high-risk groups of TRIM68 and DYNLL2 were higher than those in their respective low-risk groups (P-value < 0.001). On the other hand, the median gene expression in the low-risk group of PIKFYVE was higher than that in its high-risk group (P-value < 0.001). The principal components analysis (**Figure 5F**) revealed that the alignment of patients in the low-risk group was mostly located on the left side of the PC1 coordinate, while the alignment of patients in the high-risk group was mostly located on the right side of the PC1 coordinate, with both groups clearly distinguishable from each other.

### Survival Analysis

First, the Kaplan-Meier survival curves were plotted based on the high and low expressions of each of the three genes used for constructing the model. As seen in **Figures 5A, C**, high expression of DYNLL2 and TRIM68 resulted in higher 5-year survival in patients with osteosarcoma compared to low expression (P-value = 0.023, P-value = 0.091, respectively), whereas patients with high expression of PIKFYVE had lower survival compared to patients with low expression (P-value = 0.019, **Figure 5C**). The patients were then divided into high-and low-risk groups as predicted by the prognostic model. As visible in **Figure 5D**, the survival rate of osteosarcoma patients in the high-risk group was much lower than that in the low-risk group (P-value < 0.001).

### Diagnostic Curve ROC

**Figure 6:**
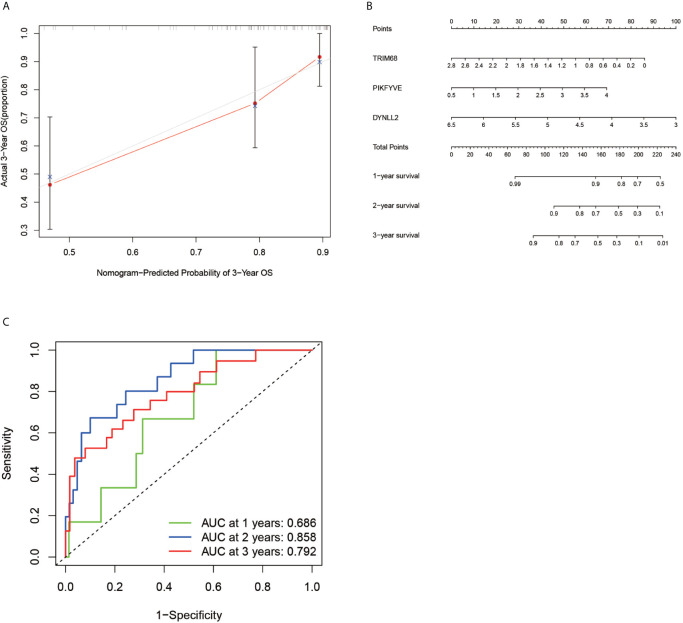
Calibration plots, column line plots and ROC diagnostic curves. Plots **(A, B)** show the calibration plots of the model and the column line plots of the predicted prognosis, respectively. Plots **(C)** demonstrates the ROC curve plot for predicting prognostic accuracy, with all areas under the curve above 50%.

According to the ROC curve (**Figure 6C**), the area under the ROC curve remained >0.5 regardless of the predicted survival time (1-year predicted survival, 2-year survival, or 3-year survival), further confirming the accuracy of the constructed model.

### Risk Map Presentation

**Figure 7:**
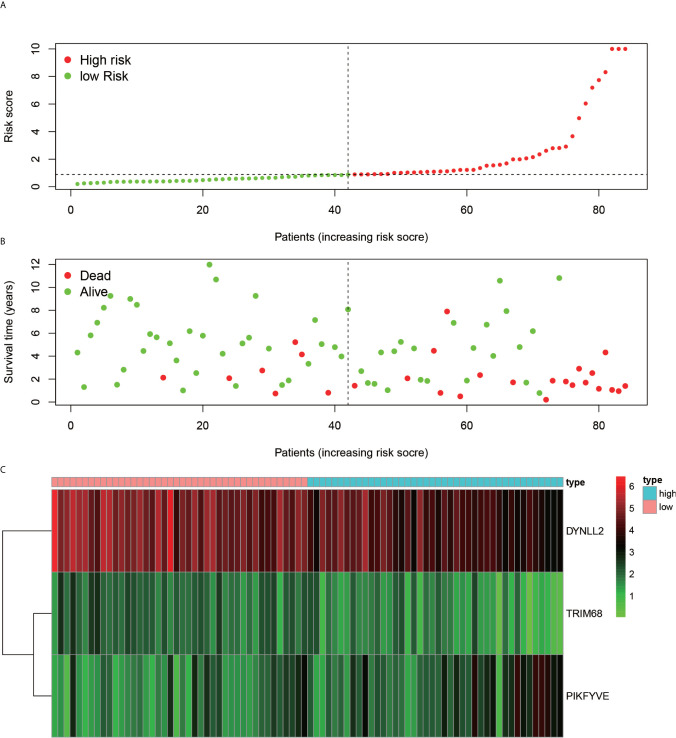
Risk diagram. **(A)** indicates that patients are ranked in descending order according to their highest risk. **(B)** indicates the time and risk of death for each patient. **(C)** indicates the expression of the three genes that were modeled in each sample.

Using the constructed model, the risk values for each patient were calculated and ranked in increasing order (**Figure 7A**). As inferred from **Figure 7B**, the patients in the low-risk group generally survived longer than those in the high-risk group. According to the risk heat map (**Figure 7C**), the risk value of both DYNLL2 and PIKFYVE increased from low risk to high risk, while that of TRIM68 decreased from low to high risk.

### Calibration and Alignment Diagrams

In order to verify the accuracy of the constructed prognostic model, a calibration diagram (**Figure 6A**) was constructed, which revealed that the predicted starting point was slightly lower than the actual starting point and the predicted focus coincided with the actual endpoint. A line graph (**Figure 6B**) was used for predicting the patient’s 1-year survival, 2-year survival, and 3-year survival based on the sum of the individual values of the indicators in the graph.

### Heat Map of the Immune Cell Composition and Correlation of the Immune Cell Composition and Model Genes for Each Sample

**Figure 8:**
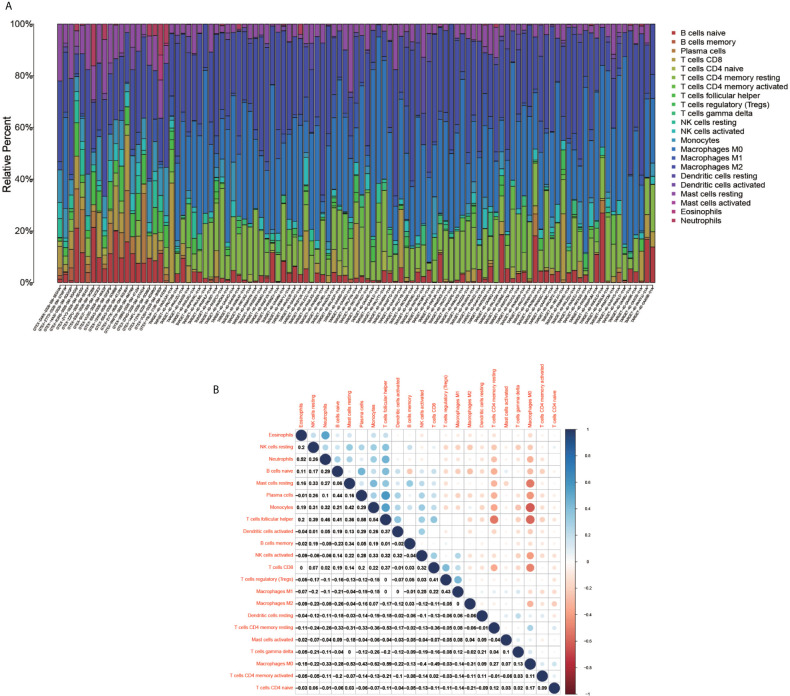
Immune cell composition diagram and correlation heat map. **(A)** indicates Histogram showing the composition of the immune cells in each sample. **(B)** indicates the heat map showing the correlation between individual immune cells, with darker red indicating a stronger positive correlation and darker blue indicating a stronger negative correlation.

**Figure 9:**
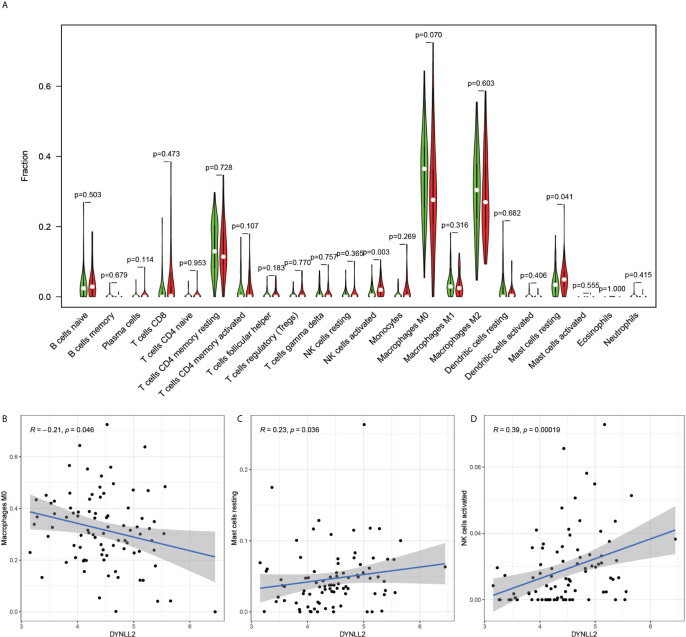
Immune cell violin plots and correlation plots of DYNLL2. **(A)** indicates the differences in the composition of the 22 immune cells based on the DYNLL2 gene. **(B–D)** show correlation plots of DYNLL2 with three different immune cells.

**Figure 10:**
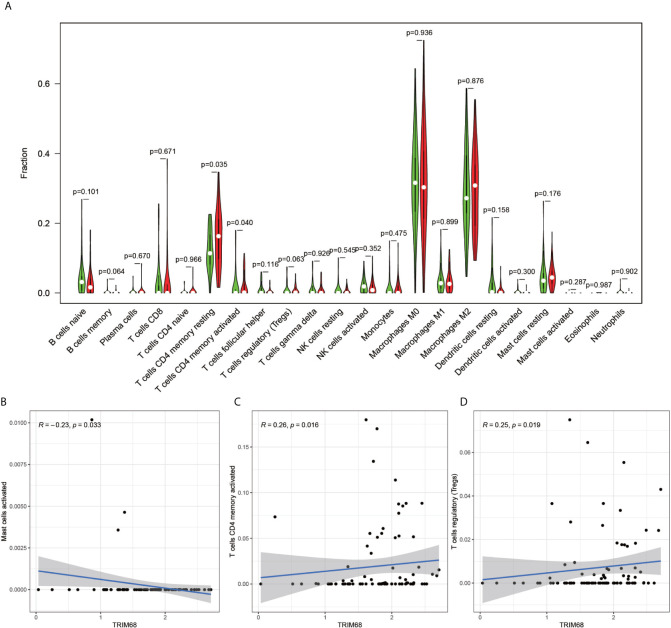
PIKFYVE immune cell violin plots and correlation plots. **(A)** indicates the differences in the composition of the 22 immune cells based on the PIKFYVE gene. **(B–D)** show correlation plots of DYNLL2 with three different immune cells.

**Figure 11:**
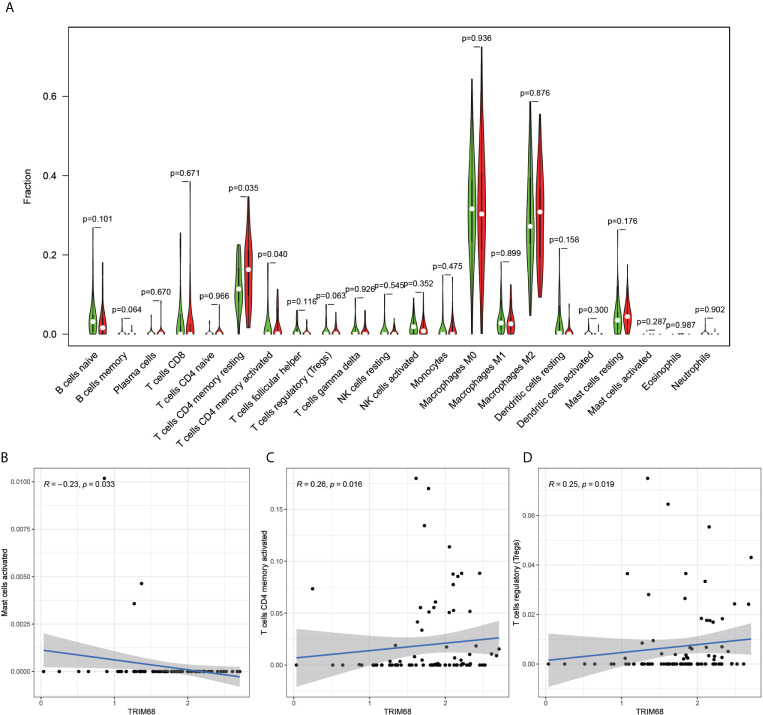
Immune cell violin plots and correlation plots of TRIM68. **(A)** indicates the differences in the composition of the 22 immune cells based on the TRIM68 gene are indicated. **(B–D)** show correlation plots of TRIM68 with three different immune cells.

The immune cell composition analysis of the gene expression matrix was performed using the CIBERSORT software, and it was revealed that each sample comprised 22 types of immune cells. The constituent plots (**Figure 8A**) and correlation heat maps (**Figure 8B**) were plotted for the samples, which presented a statistically significant p-value of <0.05. Among these, 107 samples presented statistical significance in **Figure 10A**, including 20 normal samples and 87 tumor samples. The DYNLL2-based violin plot of immune cell composition (**Figure 9A**) revealed that macrophage M0, resting mast cells, and activated NK cells were statistically significant (P-value < 0.05). The expression of the DYNLL2 gene was negatively correlated with macrophage M0 (**Figure 9B**, R = −0.21, P-value = 0.046), while it was positively correlated with both resting mast cells and activated NK cells (**Figures 9C, D**, R = 0.23 and R = 0.39, respectively). The violin diagram of immune cell composition for PIKFYVE (**Figures 10A–D**) revealed that CD8 T cells, activated memory CD4 T cells, follicular helper T cells, and regulatory T cells (Tregs) were statistically significant (P-value < 0.05) and demonstrated a trend toward negative correlation (R < 0). According to **Figures 11A–D**, activated memory CD4 T cells, regulatory T cells (Tregs), and activated mast cells were statistically significant (p-value < 0.05) for TRIM68, which demonstrated a negative correlation with cell activation (R < 0) and a positive correlation (R > 0) with activated memory CD4 T cells and regulatory T cells (Tregs).

### Real-Time Quantitative Reverse Transcription PCR (qRT-PCR)

**Figure 12:**
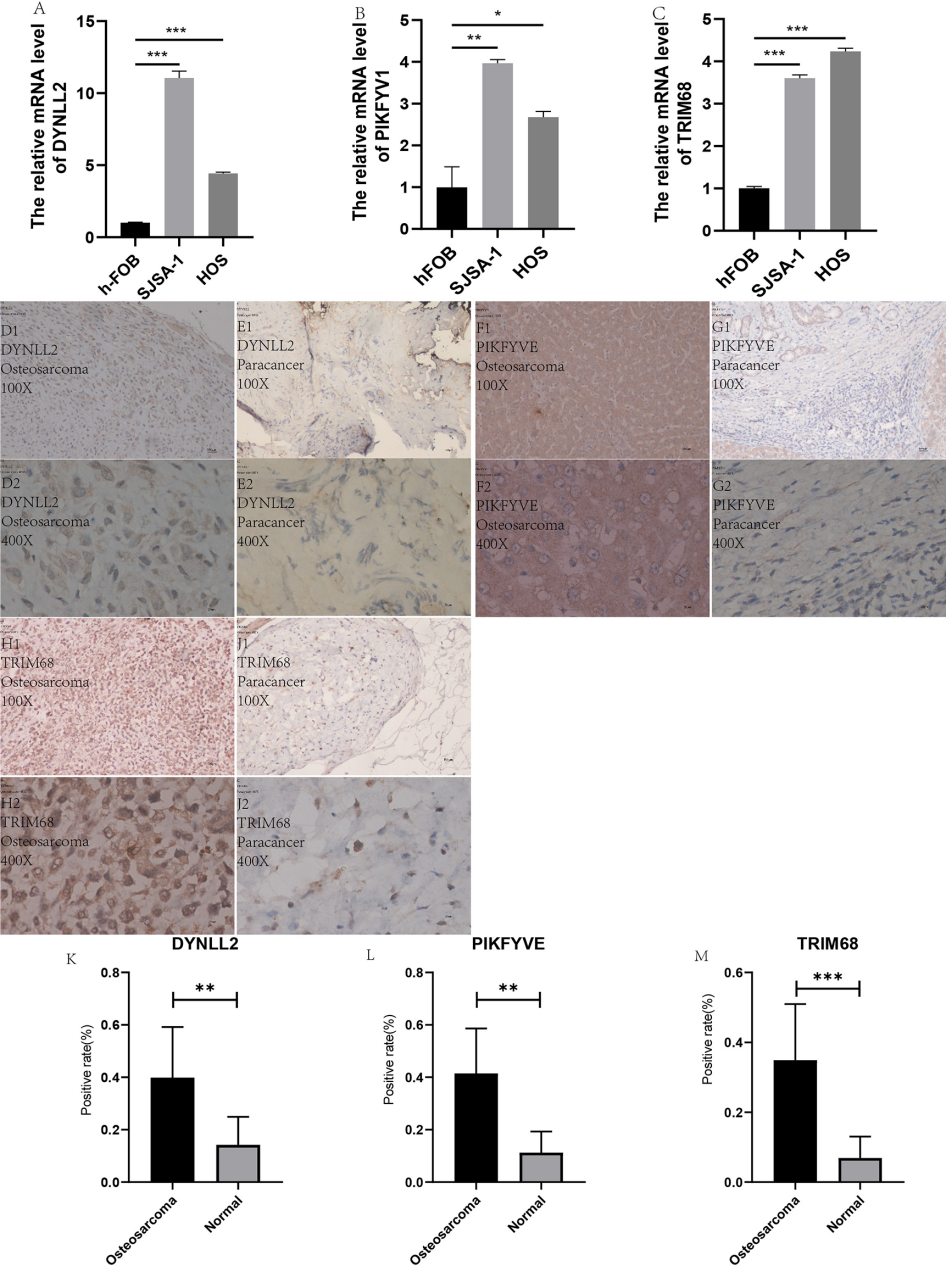
qRT-PCR and immunohistology. **(A–C)** plots show the expression of these three genes in two types of osteosarcoma cells as well as in normal control cells, respectively. **(D1–J2)** plots demonstrate the expression of these three genes in osteosarcoma and paraneoplastic tissues (guide inverted microscopy 100×). The **(K–M)** plots represent the statistics of the positivity rate of these three genes in pathological sections, respectively. **(J)** has been used to number the immunohistochemically stained images. “*” represents P < 0.05, “**” represents P < 0.01, “***” represents P < 0.001.

In order to verify the accuracy of the results, we performed in vitro laboratory cell experiments. We extracted RNA from the three cell lines and after several experimental steps, the final qRT-PCR results showed that the results identified significant differences in the expression of TRIM68, PIKFYVE and DYNLL2 genes in normal human osteoblast cell lines (hFOB1. 19) and OS cell lines (SJSA-1 and HOS) (**Figures 12A–C** **)**. The graph shows that the expression of these three genes was significantly higher in both osteosarcoma strains than in normal control cells. This result also concords well with the results of our previous analysis.

### Immunohistochemistry

All immunohistological images were observed under an inverted microscope, and the staining differences were compared between osteosarcoma specimens and normal control tissue specimens. We performed immunohistological staining analysis on 36 pathological tissue sections for six pairs (osteosarcoma and paraneoplastic tissue) for each gene, and the positive rate of staining was calculated for all images using ImageJ software. The three antibodies we purchased are all specific antibodies against these three genes and do not respond to immune cell infiltration. Therefore, the positive areas in the pictures are the result of specific antigen antibody reactions (**Figures 12D1–J2**) and are not related to immune cell infiltration. We then statistically analyzed the positive rate of each gene in osteosarcoma and paraneoplastic tissues by means of the paired sample mean t-test in IBM SPSS Statistics 25, and found a statistically significant difference in the immunohistological positive rate of each gene in osteosarcoma and paraneoplastic tissues with a P-value < 0.05. We found from the immunohistological images that all three genes were significantly more expressed in osteosarcoma than in paraneoplastic tissue (**Figures 12D1–J2**). Our statistical analysis of the positive rates of all immunohistology graphs revealed that the positive rates of immunohistological staining for all three genes were higher in osteosarcoma than in paraneoplastic tissue (**Figures 12K–M**).

## Discussion

In the present study, GO enrichment analysis and KEGG pathway enrichment analysis were performed for autophagy-related genes, and it was observed that the GO entries were distributed mainly in autophagy, a process utilizing autophagic mechanism and regulation of autophagy. Autophagy, which plays an essential role in tumor development by operating cell-autonomous mechanisms in cancer cells, is also a vital component of the innate immune response, as evidenced by an increasing number of studies (24). Furthermore, autophagy expression is reported to be elevated in pancreatic ductal carcinoma and may promote tumor growth (25). Recent studies have demonstrated that the role of autophagy depends on the environment, i.e., autophagy in tumor cells may both promote the tumor progression and inhibit tumorigenesis (26). On the other hand, the KEGG pathway analysis in the present study revealed that the autophagy-related gene pathway was enriched mainly in the phagosome, autophagy-animal, and mTOR signaling pathways. As an essential cellular mechanism that provides for a variety of cellular needs, autophagy not only disrupts the homeostasis in the body but also causes various diseases, including cancer (27). Interestingly, the mTOR signaling pathway is reported to play a critical role in the proliferation, development, and progression of human ovarian cancer (28). Besides ovarian cancer, this pathway is also reported in colorectal cancer, with the genes associated with colorectal cancer-inhibiting the proliferation of colorectal cancer cells and promoting apoptosis through the inhibition of the mTOR signaling pathway (29). All these findings are consistent with our study, in which three autophagy-related genes TRIM68, PIKFYVE, and DYNLL2 were used for constructing a prognostic model, and the mortality rate of the patients in the high-risk group was observed to be much lower than that in the low-risk group. In addition, to delineate high-and low-risk groups, the different principal component analysis (PCA) 3D plots were generated in the present study.

The current research on TRIM68 is not adequate. It is reported that certain members of the TRIM protein family can act as cancer regulators, leading to tumor development and progression (30). Interestingly, tumor cells have been demonstrated to damage the immune cells in the tumor microenvironment and evade the surveillance role of immune cells, which is vital in tumor cell development (31). This is consistent with our findings, as well. A high volume of research has been carried on PIKFYVE in cancer. Studies have demonstrated that PIKFYVE activity protects the Ras mutant cells from starvation-induced cell death during nutrient depletion and also supports the proliferation of these cells (32). Interestingly, in our study, PIKFYVE exhibited a negative correlation with CD8 T cells, activated memory CD4 T cells, follicular helper T cells, and regulatory T cells, all of which play important roles in monitoring tumor progression (33–37). Previous studies have demonstrated that DYNLL2 is a key gene in hepatocellular carcinoma and plays an integral role in cancer development (38). In the present study, DYNLL2 exhibited a significant negative correlation trend with macrophage M0, which are the most abundant cells in stage N1 tumors (39). This implied that with extended tumor development, there would be fewer macrophages M0. Our study shows that in osteosarcoma, the three autophagy-related genes that build the model are closely associated with different immune cells, and on the other hand immune imbalance in tumors is an important component of tumor development.

In the present study, univariate Cox regression analysis, multivariate Cox regression analysis, and LASSO regression analysis were used for constructing a predictive model for osteosarcoma prognosis using the gene expression matrix data from the UCSC Xena and GTEx databases along with the corresponding clinical information data. The accuracy of the constructed model was verified using the principal components analysis, Kaplan-Meier survival analysis, ROC diagnostic curve analysis, risk prediction analysis, line graphs, and calibration plots. Subsequently, component analysis of immune-associated genes was performed by using the CIBERSORT software on the expression matrix, which revealed that each of the three genes used for constructing the model correlated strongly with certain immune cells. In conclusion, an accurate prognostic model for osteosarcoma was constructed using a fairly sophisticated analytical approach, along with the immune cell composition analysis of the genes used for constructing the model. In addition, our analysis was experimentally verified by using qRT-PCR to detect the expressions of TRIM68, PIKFYVE, and DYNLL2 genes in a normal human osteoblast cell line (hFOB1.19) and OS cell lines (SJSA-1 and HOS). It was observed that the expression of the DYNLL2 PIKFV1 and TRIM68 gene was significantly higher in two cell lines than in normal human osteoblast cell lines. We also performed immunohistological studies in human pathological tissue sections, which showed that the expression of these three genes was significantly higher in osteosarcoma than in paraneoplastic tissue. We have demonstrated the reliability of our analysis through laboratory validation at the cellular level and laboratory validation of human tissue immunomics. All evidence from our study demonstrates that TRIM68, PIKFYVE, and DYNLL2 are high-risk genes involved in the development of osteosarcoma and can be used as biomarkers for predicting the prognosis of osteosarcoma.

As with all research, our study also has certain limitations. First, the sample size was inadequate, with only 88 osteosarcoma samples and 396 normal samples included in the study, which is far from what can be considered an adequately large sample set. Second, the issue of tumor typing, i.e., the specific typing of each tumor is different and thus leads to a different clinical outcome, was not considered in the present study.

## Conclusions

TRIM68, PIKFYVE, and DYNLL2 are high-risk genes involved in the development of osteosarcoma and can be used as possible biomarkers for predicting the prognosis of osteosarcoma.

## Data Availability Statement

The original contributions presented in the study are included in the article/supplementary material. Further inquiries can be directed to the corresponding authors.

## Ethics Statement

The studies involving human participants were reviewed and approved by the Ethics Department of the First Clinical Hospital of Guangxi Medical University. Written informed consent for participation was not required for this study in accordance with the national legislation and the institutional requirements.

## Author Contributions

JJ, CL, and XZ designed the study. GX, TL, SL, and CY analyzed the data. ZZ, ZL, ZW, JC, TC, and HL were in charge of digital visualization. JJ wrote and revised the manuscript. CL and XZ revised the manuscript. All authors contributed to the article and approved the submitted version.

## Funding

This study was supported by the Youth Science Foundation of Guangxi Medical University, Grant/Award Numbers: GXMUYFY201712; Guangxi Young and Middle-aged Teacher’s Basic Ability Promoting Project, Grant/Award Number: 2019KY0119; and National Natural Science Foundation of China, Grant/Award Numbers: 81560359, 81860393.

## Conflict of Interest

The authors declare that the research was conducted in the absence of any commercial or financial relationships that could be construed as a potential conflict of interest.

## Abbreviations

DEGs, differentially expressed genes; GO, gene ontology; CTL, CD8+ T cytotoxic T lymphocytes; GSEA, Gene Set Enrichment Analysis; KEGG, KEGG enrichment analysis; LASSO, least absolute shrinkage and selection operator.
